# Process Development of Polyhydroxyalkanoates Production by Halophiles Valorising Food Waste

**DOI:** 10.3390/bioengineering9110630

**Published:** 2022-11-01

**Authors:** Ke Wang, Chang Chen, Ruihong Zhang

**Affiliations:** Biological and Agricultural Engineering Department, University of California Davis, One Shields Avenue, Davis, CA 95616, USA

**Keywords:** process development, polyhydroxyalkanoates, food waste, spent salts recycling, *Haloferax mediterranei*

## Abstract

Polyhydroxyalkanoates (PHA) is an emerging biodegradable plastic family that can replace a broad spectrum of conventional thermoplastics and is promising in the sustainable process development and valorization of organic waste. This study established a novel production system of PHA from food waste through halophilic microbial fermentation with spent medium recycling. The essential processing parameters for batch cultivation of *Haloferax mediterranei* were optimized for food waste substrate (a 40 g/L loading and 2.5 vvm of aeration), which achieved a yield of 0.3 g PHA/g COD consumed. A batch bioreactor system was developed, which produced 7.0 ± 0.7 g/L cell dry mass and 4.5 ± 0.2 g/L PHA with a 20% dissolved oxygen (DO) level. A DO above 50% saturation resulted in faster cell growth and similar cell mass production but 25% less PHA production. The spent saline medium, treated with H_2_O_2_ and rotary evaporation, was successfully reused for four consecutive batches and provided consistent PHA concentrations and product qualities.

## 1. Introduction

Polyhydroxyalkanoates (PHA) is a family of high-value polyesters with properties similar to thermoplastics and is one major material used for manufacturing biodegradable plastics [1]. PHA is commercially available for various applications, including packaging, medical materials, nutritional supplements, and biofuels [2]. The high production cost is a major barrier to market expansion [3]. There is a study that found that feedstock cost contributes over 40% of the total annual production cost [4]. Food waste is a potential low-cost feedstock because of rich nutrients which provide essential carbon, nitrogen, and phosphorous sources for microbial cultivation and PHA production [5]. The complex organics of municipal food waste often require pretreatment approaches to generate precursors for PHA synthesis [3]. Acidogenic fermentation has been widely applied to commercial anaerobic digesters to process food waste. After fermentation, most studies endorsed mixed microbial consortium (MMC) to produce PHA [6,7,8,9]. Studies on utilizing pure culture PHA production from fermented food waste have been scarce. An extreme halophilic archaeon, *Haloferax mediterranei*, can metabolize various types of waste feedstocks [5,10,11] and requires a high saline environment (19% total salts) which minimizes the risk of contamination and facilitates an extraction process through osmotic shock [12], it is, therefore, a promising candidate. The PHA produced by *H. mediterranei* is characterized as poly (3-hydroxybutyrate-co-3-hydroxyvalerate) (PHBV), which is a high-value PHA and has various applications.

The critical parameters involved in PHA production, including feedstock type, substrate loading, and aeration rate, have not been fully understood for this halophilic microorganism. The nutrients derived from food waste can vary even in the same anaerobic digester. High substrate loadings with high concentrations of volatile fatty acids (VFA) may also inhibit cell growth [13]. Another study reported that the substrate loadings of seaweed hydrolysate significantly impacted cell mass and PHA production by *H. mediterranei* [14]. The saturated dissolved oxygen (DO) in a medium with 19% total salts is 2.50 mg/L at 37 °C, which is only 37% of the saturated DO (6.73 mg/L) in freshwater [15]. Therefore, the aeration rate can be critical to *H. mediterranei*. There are no universal guidelines for DO level in culturing this halophile in the literature. Some studies used shaking flasks in the ambient atmosphere [16,17]. Some used various-sized bioreactors with forced aeration: a 100% DO saturation during the entire culturing period [18]; a 50% DO saturation was maintained during balanced cell growth, and the DO dropped to 30% for PHA formation process [19]; others maintained a 20% saturated DO [17,20,21]. Therefore, this study intended to determine the optimum levels of these key processing parameters in halophilic PHBV production from fermented food waste and understand the variance between production scales.

Previous studies on *H. mediterranei* mostly did not consider the treatment of spent salts after production. Salts are the principle water pollutants in freshwater bodies [22], and wastewater with chloride ions exceeding 1500 mg/L cannot be discharged [23]. Therefore, treating spent brine in large-scale productions would be economically critical. One study proposed a method of using a hot decanoic acid solution to precipitate salts from the spent stillage medium [24]. However, it requires a long processing time which adds to the operating cost. Another study reported that direct reuse of the spent supernatant resulted in a 68% lower final PHA production than the original batch [20]. Therefore, there is an urgent need to develop an efficient technique to recycle the spent salts with high productivity and low processing cost. 

Another hotspot that has not been well studied is the impact of the leftover nutrients that accumulate after multiple recycled batches on cell growth and production yield. These compounds include residual sugars, short-chain carboxylates, micronutrients that are not fully consumed by *H. mediterranei*, and cell metabolites generated during cell growth, such as acetic acid and extracellular polysaccharides (EPS). Those leftover nutrients can contribute substantially to organics after multiple batch runs, which may influence cell growth and PHBV production. Chemical oxidation by H_2_O_2,_ which is widely used in industrial wastewater treatment [25,26], can be effective in removing excessive chemical oxygen demand (COD) from the spent saline medium. H_2_O_2_ is also known as the oxygen supplier to microorganisms in biological treatment facilities due to its natural decomposition into oxygen and water [26]. 

Therefore, this study proposed to develop a novel and sustainable bioconversion system of halophilic PHA production integrating the utilization of municipal food waste, with three main objectives: (1) to determine the optimum processing parameters that provide the highest PHA production from food waste; (2) to design and develop a robust integrated bioreactor system; and (3) to develop an efficient and applicable technique to process and recycle the spent salts while maintaining consistent production and product qualities.

## 2. Materials and Methods

### 2.1. Feedstock Collection and Pretreatment

The feedstock was collected from the Renewable Energy Anaerobic Digester (READ) (Davis, CA, USA), which processes municipal food waste from local grocery stores and dining commons. READ employs a two-stage high-solid anaerobic fermentation/digestion technology consisting of a fermentation process to decompose food waste into short-chain carboxylic acids, including lactic acid (La), acetic acid (Ac), propionic acid (Pr), butyric acid (Bu) and other volatile fatty acids (VFA), soluble nitrogen, phosphorous, and micronutrients; and a digestion process where those nutrients from the previous stage are further converted into biogas [27]. The feedstock used in this study was the fermented food waste collected from the first stage. Prior to collection, the feedstock samples were treated by an onsite screw press to pass through a 1-mm screen; as a result, a thin slurry was recovered after the removal of most of the large particles.

**Figure 1:**
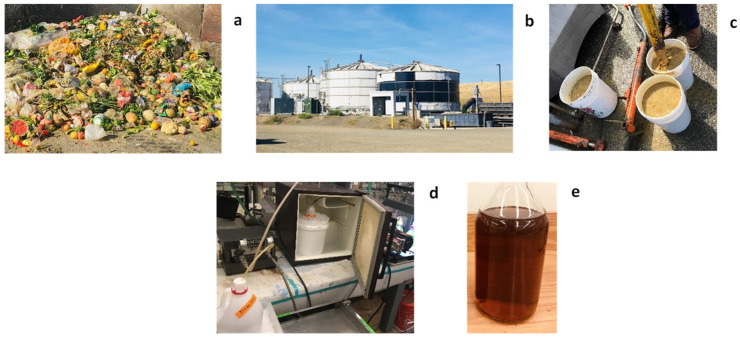
Feedstock collection and pretreatment: (**a**) Municipal food waste at the READ facilities; (**b**) READ facilities; (**c**) Thin slurry of fermented food waste; (**d**) Bench-scale crossflow microfiltration unit; (**e**) Fermented food waste permeates after filtration.

Two feedstock samples with a volume of 45 L each were collected at two different times of the year. The samples were transported immediately after collection and stored in a −10 °C freezer until further use. The feedstock samples were processed through a bench-scale crossflow microfiltration unit (Sterlitech, Auburn, WA, USA) with a 0.22 μm flat-sheet membrane (Synder, Vacaville, CA, USA) to further remove suspended solids. The temperature was maintained at around 4 °C by a refrigeration unit and ice bath during the filtration process to minimize the loss of volatile solids. An outflow stream free of suspended particles, also known as the fermented food waste permeates (FWP), was used as the sole substrate for the following PHA production experiments, without any other carbon or nitrogen sources for cell cultivation. The processes of feedstock collection and pretreatment are shown in Figure 1.

### 2.2. Microorganism and Culture Conditions

The wild-type halophilic archaeon, *H. mediterranei* (ATCC 33500, Manassas, VA, USA), was used as the microbial PHA producer. The strain was firstly grown in the standard seed medium (containing NaCl, 156 g/L; MgCl_2_·6H_2_O, 13 g/L; MgSO_4_, 9.8 g/L; CaCl_2_·6H_2_O, 1 g/L; KCl, 4 g/L; NaBr, 0.5 g/L, yeast extract, 5 g/L, glucose, 10 g/L, and NaHCO_3_, 2.1 g/L) at 37 °C until the late exponential phase. The cell culture was centrifuged at 2940× *g* for 5 min and resuspended in the minimum salt medium (MSM) (containing NaCl, 156 g/L; MgCl_2_·6H_2_O, 13 g/L; MgSO_4_, 9.8 g/L; CaCl_2_·6H_2_O, 1 g/L; KCl, 4 g/L; NaBr, 0.5 g/L and FeCl_3,_ 5 mg/L with 10 mL/L trace metal solution SL-6, containing ZnSO_4_·7H_2_O, 100 mg/L; MnCl_2_·4H_2_O, 30 mg/L; H_3_BO_3_, 300 mg/L; CoCl_2_·6H_2_O, 200 mg/L; CuCl_2_·2H_2_O, 10 mg/L; NiCl_2_·6H_2_0, 20 mg/L; Na_2_MoO_4_·H_2_O, 30 mg/L) [11,28]. The cell suspension was used as the inoculum for all experiments. All the chemicals were purchased from Thermo Fisher Scientific (Waltham, MA, USA).

The two FWP feedstock samples, named FWP 1 and FWP 2, were used as the sole substrate to cultivate *H. mediterranei*. Two sets of two-factor factorial experiments were conducted: (1) three levels of aeration rate (in air volume per working volume per minute, vvm): 0.5, 1.5, 2.5 and two levels of FWP 1 loading (in g/L COD): 40, 80; (2) two types of substrate: FWP 1, FWP 2 and four levels of substrate loading (in g/L COD): 20, 40, 60, 80. The experiments were conducted using 250-mL glass bioreactors with 80% working volume. The bioreactors were housed in an incubator with a temperature of 37 ± 0.5 °C. Forced aeration was applied to all reactors using air pumps, and the aeration rate was adjusted using air flowmeters. The air was pre-humidified by water to leverage volume loss by forced aeration [5]. MSM was added to create a salinity at 188 ± 2 parts per thousand (ppt). The same inoculation was applied for all reactors to give an initial optical density (OD) of 1.0 ± 0.2 at 520 nm wavelength. The buffer solution (50 mM NaHCO_3_) was added at the beginning of the experiments. The pH was maintained at 7 ± 0.2 throughout the cell cultivation through adjustment by either 3 M NaOH solution or 3 M HCl solution. The dissolved oxygen (DO) levels of cell broth were monitored daily by a DO meter (YSI ProDSS, Rye Brook, New York, NY, USA). The cells were cultured for 120 to 216 h until cell growth reached the stationary phase. 

### 2.3. Batch Production System with Two Aeration Strategies

**Figure 2:**
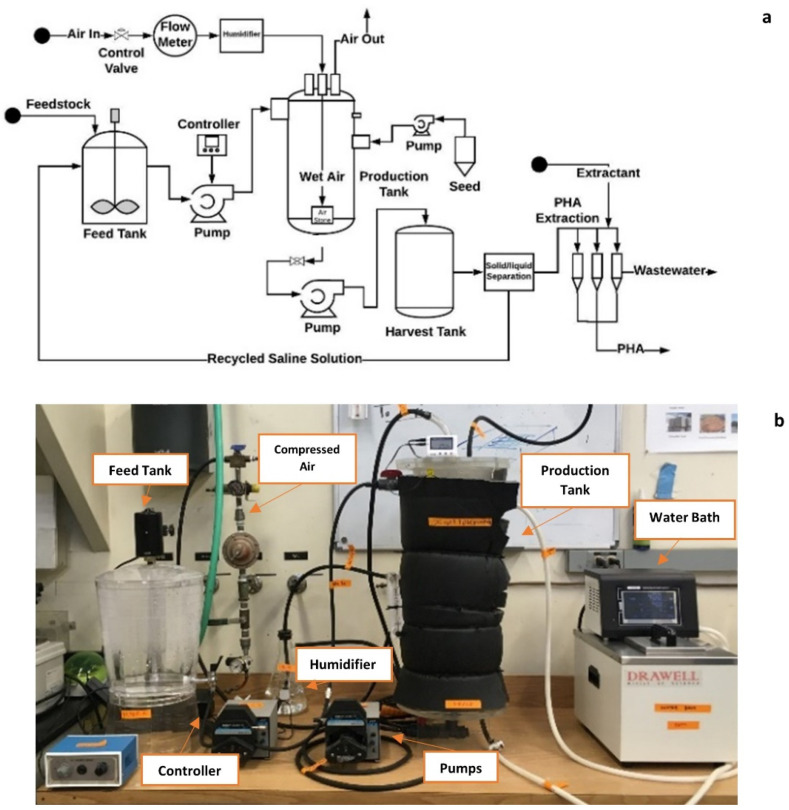
The 6-L integrated bioreactor system for PHBV production: (**a**) Schematic design; and (**b**) the actual production system after construction.

A bench-top bioreactor system was developed for PHA production from FWP (shown in Figure 2). The system consists of a 6-L feeder tank with overhead mixer; a peristaltic flow pump with flowrate controller (Grainger, Bakersfield, CA, USA); a 6-L aerated reactor with a controlled air sparging at the bottom (by compressed air outlet, a humidifier to moisten the air, and an inline air flowmeter) and temperature control (using a water jacket and insolation); a circulating water bath (equipped with internal pumping and heating-cooling components) (Drainwell, Shanghai, China); a peristaltic pump with controlled flowrate for inoculation (Grainger, Bakersfield, CA, USA); a 6-L harvest tank; an external centrifuge (Heraeus Multifuge X1R, Thermo Fisher Scientific, Waltham, MA, USA) for solid/liquid separation; a PHA extraction/purification block including consistent temperature control and rotation; and built-in data logging units of temperature, pH, and DO. All bioreactors were made of plastic materials to prevent high salinity corrosion.

Batch production experiments were conducted endorsing the optimum cell culture conditions, including substrate type, substrate loading, and aeration rate, which were determined from previous experiments. The working volume was 5 L. Other culturing conditions were the same as those described in previous sessions. Four production batches were operated with different aeration strategies where (1) the aeration rate was controlled at 2.5 vvm in the first two batches and (2) the DO level of cell broth was controlled above 50% of the saturation level in the last two batches with variable aeration rates of 2.5 to 8 vvm. The cell growth, PHA production, substrate consumption, DO level, pH, and temperature were monitored daily during the batch period. The cells were cultured for 132 h until cell growth reached the stationary phase. Duplicates were prepared for each of the variable conditions.

### 2.4. Spent Saline Medium Treatment and Recycling

The FWP 1 was used as the substrate to provide a soluble COD of 20 g/L. The 250-mL bioreactors with a working volume of 200 mL were used for cell cultivation experiments. The cell culturing conditions were the same as described previously in Session 2.2. A total of four consecutive batch runs were conducted, with the spent salts being recycled and reused for cell cultivation. At the end of each batch run, the cells were separated from the spent medium through centrifugation at 2940× *g* for 30 min. The spent saline medium (SSM) from the second batch was treated with H_2_O_2_ by adding an aqueous solution containing 30% H_2_O_2_ at 5% of the total volume and reacting for 8 h. After treatment, the SSM was processed by a rotary evaporator (Büchi R-3, Switzerland). The evaporation was conducted with an applied vacuum and a temperature of 57 °C for 30 min. The evaporation treatment was stopped when salt crystals started appearing in the brine. The remaining brine solution was then used for cell cultivation in the following batch runs with two mass levels: 80% and 90% of the total spent salt mass being recycled. The same conditions of nutrients and cell cultivation were provided to all batch runs.

### 2.5. Analytical Methods

#### 2.5.1. Feedstock Characterization

Before and after the microfiltration process, 10 mL of homogeneous samples were collected from the inflow, permeate, and retentate streams and characterized for the composition of the major nutrients. The concentrations of short-chain carboxylic acids, including La and VFAs, were measured by high-performance liquid chromatography (HPLC) (Agilent, Santa Clara, CA, USA) following an analytical method described by [29]. COD, total nitrogen, and reactive phosphorous contents were measured by standard chemical kits (HACH, Loveland, CO, USA). The trace elements and micronutrients, including K, Ca, Mg, Na, B, Zn, Cu, Mn, Fe, and Ni, were measured by UC Davis Analytical Lab using a nitric acid/hydrogen peroxide microwave digestion method. 

#### 2.5.2. Cell Biomass Measurement

Cell growth was monitored by collecting 2 mL of strain broth, and its OD was measured by a spectrophotometer (Thermo Fisher Scientific, Waltham, MA, USA) at a wavelength of 520 nm [18]. The cell dry mass (CDM) was determined as the volatile suspended solids (VSS) of the cell broth: 10 mL of cell broth was sampled and subjected to centrifugation at 2940× *g* for 20 min. The cell pellet was washed with MSM and measured following a standard method [30]. As for the measurement of nutrients, the supernatant from the CDM measurement was filtered through a 0.22 μm membrane. The filtrate was measured for the COD, total N, reactive P, and the contents of La and VFAs using the methods described in the previous session.

#### 2.5.3. Product Extraction and Quantification

PHA was extracted and quantified following a method by [31] with modifications: 40 mL cell broth was collected at designated time points and treated by immediate centrifugation at 2940× *g* for 30 min. The cell pellet was washed twice with 0.1% sodium dodecyl sulfate (SDS) solution and then deionized water. The washed pellet was first dissolved in 2 mL dichloromethane and 2 mL acidic methanol (3% *v*/*v* H_2_SO_4_), with 1 g/L benzoic acid as the internal standard. The liquid mixture was then heated in a digital reactor block (HACH DRB200, Loveland, CO, USA) at 105 °C for 4 h and cooled to room temperature. The solution was added with 1 mL of deionized water, mixed, and settled until a phase separation was observed. The organic phase was then transferred to a clean vial for quantification. Poly (3-hydroxybutyric acid-co-3-hydroxyvaleric acid) with 12% HV (Sigma-Aldrich, Burlington, MA, USA) was used as the standard PHA chemical. The samples were analyzed using a gas chromatography (GC) (Agilent, Santa Clara, CA, USA) method described in a previous research article by [5].

### 2.6. Statistical Analysis

All the experiments were conducted in triplicates, and the results are presented as mean ± standard deviation (SD). Two-way analysis of variance (ANOVA) with a post-hoc Tukey test was performed to provide the significance of the difference between mean values for comparison. The significance level (α) was 0.05 throughout the study. The software Origin Pro (OriginLab, Northampton, MA, USA) was used for all data analysis in this study.

## 3. Results

### 3.1. Characterization of Microfiltration Streams

The fermented food waste feedstock collected from READ was pre-treated by a microfiltration process to obtain a particle-free FWP. The FWP accounted for 73 ± 3% (*v*/*v*) of the initial feedstock inflow and was used as the substrate for cell cultivation and PHA production by *H. mediterranei*. The rest of the inflow came out as the retentate containing the suspended solids, which the microbes cannot utilize. Instead, it could be used as seed culture for anaerobic fermentation of food waste or recycled back to READ facilities for further anaerobic digestion to generate biogas as a source of bioenergy.

Table 1 compares the nutrient composition of influent, permeate (FWP), and retentate of the two fermented food waste feedstocks. The three streams of the same feedstock had close contents of short-chain carboxylic acids. However, the concentrations of La, Ac, Pr, and Bu in both permeate and retentate were slightly lower than those in the inflow stream. This may be due to the loss of volatile organic compounds (VOC) during microfiltration. The loss can be reduced by increasing the filtration flux and shortening the processing time. Among the three filtration streams, inflow and retentate had higher COD, total N, and reactive P concentrations than permeate because permeate contains only the soluble nutrients of the inflow. The suspended solids of inflow were mainly food waste scraps with a small portion of microbial biomass, which can be further fermented into soluble nutrients and utilized for PHA production. Permeate 2 (FWP 2) had more La and Ac than permeate 1 (FWP 1), and FWP 1 had more Pr than FWP 2. The carboxylic acids accounted for 67% of the total COD in FWP 1 and 92% of the total COD in FWP 2. The major species of short-chain carboxylic acids were La, Ac, Pr, and Bu for both permeates, with different compositions (% of total carboxylic acids): 41%, 17%, 33%, 7% in FWP 1; and 51%, 23%, 18%, and 6% in FWP 2. FWP 1 had higher total N and reactive P contents than FWP 2. Table 2 lists the results of micronutrients and trace elements in the permeate samples, suggesting that FWP 1 and FWP 2 had close contents of those compounds. Compared with the standard medium for cell culture (MSM and SL-6), both permeate samples were able to provide enough nutrients and trace elements of K, Ca, B, Zn, Cu, Mn, Fe, and Ni that are essential for cell growth. With FWP 1 and FWP 2 as the sole substrate for cell culture, only additional Na and Mg salts were needed to provide enough salinity.

### 3.2. Effect of Aeration Rate

**Figure 3:**
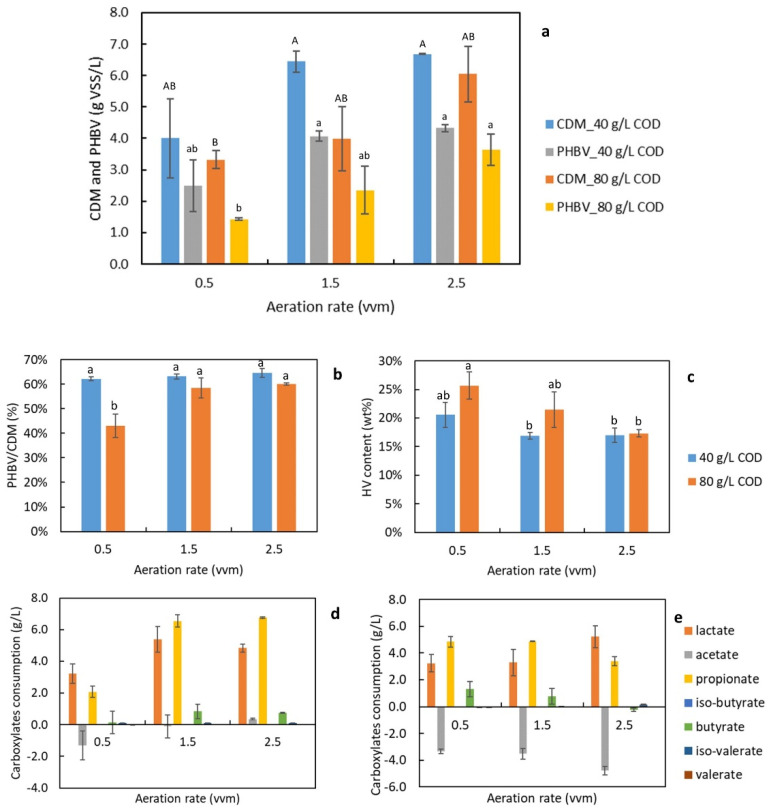
Effect of aeration rate on (**a**) final concentrations of CDM and PHBV; (**b**) cell content of PHBV; (**c**) the HV content of PHBV polymers; (**d**) the consumption of short-chain carboxylates with the low substrate loading (40 g/L COD); and (**e**) with the high substrate loading (80 g/L COD). Note: Same letters indicated that the mean values were not significantly different (*p* > 0.05).

Figure 3a demonstrates the cell mass and PHBV production with different levels of aeration rate. The aeration rate significantly affected PHBV production (*p*-values was 0.003). The interaction between the two parameters was not statistically significant (the *p*-value was 0.4). With a COD loading of 40 g/L, the lowest aeration level of 0.5 vvm gave a much lower CDM production (4.00 ± 1.25 g/L VSS) than the higher aeration levels (6.45 ± 0.34 g/L VSS from 1.5 vvm, and 6.69 ± 0.02 g/L VSS from 2.5 vvm). And the two higher aeration levels resulted in close CDM production, as mentioned previously. Low substrate loading yielded higher CDM for all aeration levels than high substrate loading. For instance, with an aeration rate of 0.5 vvm, the CDM produced from 80 g/L COD loading was 3.33 ± 0.28 g/L VSS, which is much lower than the CDM of 4.00 ± 1.25 g/L VSS produced from 40 g/L COD. This may be due to the substrate inhibition that occurred with high concentrations of VFAs in the medium [32]. The growth of *H. mediterranei* was found to decelerate with increasing loadings of Ac, Pr, Bu, and Va [11]. The trend of PHBV formation corresponded to cell growth because PHBV synthesis was a growth-associated reaction [5]. The highest CDM and PHBV production was 6.70 ± 0.02 g/L VSS and 4.3 ± 0.1 g/L, respectively, which were achieved with the highest aeration rate (2.5 vvm) and the lower substrate loading (40 g/L COD).

As illustrated in Figure 3b, the PHBV content of cell mass remained stable at 62% to 64% among all different aeration rates, except for a statistically lower PHBV content from 80 g/L COD loading and 0.5 vvm of aeration. The HV contents of the polymer (shown in Figure 3c) were not statistically different among different aeration rates for 40 g/L COD loading. Figure 3d,e demonstrates the consumption of short-chain carboxylates with different growth conditions. Among all carboxylate species, La, Pr, and Bu were the major ones consumed by *H. mediterranei*. With low substrate loading, there was more consumption of carboxylates with increasing aeration from 0.5 to 1.5 vvm, and it remained consistent between 1.5 and 2.5 vvm. For example, cells consumed 2.08 ± 0.36, 6.54 ± 0.39, and 6.76 ± 0.05 g/L Pr with increasing aeration of 0.5, 1.5, and 2.5 vvm. The consumption of acetate was negative in some cases, as shown in Figure 3e, meaning the cells produced and accumulated more acetate than consumed. This phenomenon was also observed in sugar substrates which caused a pH drop below 6 during cell culturing [14]. These results suggest that an aeration rate of 0.5 to 1.5 vvm, which is normally used in the biotech industry [33], was not sufficient for culturing *H. mediterranei*. A low saturated DO level in high-saline water also causes a low oxygen transfer rate [34]. Therefore, an aeration rate of 2.5 vvm is recommended for a bioreactor study on this halophile. 

### 3.3. Effects of Substrate Loading and Type

**Figure 4:**
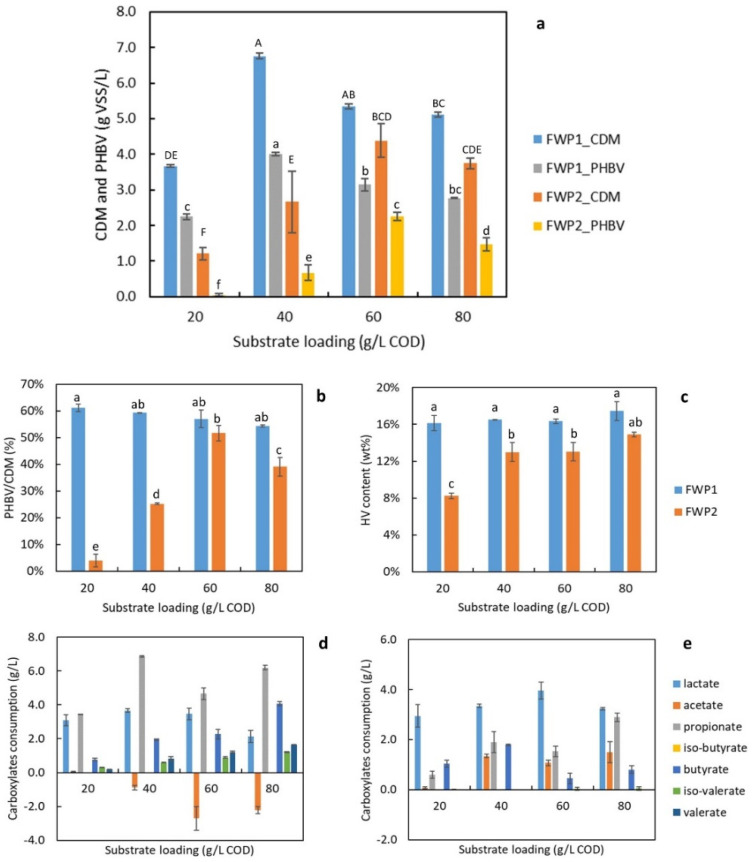
Effects of feedstock type and substrate loading on (**a**) CDM and PHBV production; (**b**) cell content of PHBV; (**c**) the HV content of PHBV; (**d**) the consumption of short-chain carboxylates with FWP 1 as feedstock; and (**e**) FWP 2 as feedstock. Note: Same letters indicated that the mean values were not significantly different (*p* > 0.05).

The effects of feedstock type and substrate loading were studied with a fixed aeration rate of 2.5 vvm. Figure 4a illustrates the concentrations of CDM and PHBV produced with different substrate loadings and feedstock types. FWP 1 and FWP 2 had quite different nutrient compositions of short-chain carboxylates, as described in Session 3.1. The substrate type and loading had significant effects on both CDM and PHBV productions (*p*-values for CDM were 1.76 × 10^−6^ and 3.93 × 10^−5^, for PHBV, were 3.14 × 10^−9^ and 1.76 × 10^−6^). The interaction between the two factors was also significant (*p*-value for CDM was 0.0012 and for PHBV was 5.66 × 10^−6^). Among all substrate loadings, the CDM produced from FWP 1 was up to 2 times more than FWP 2. For example, with a 40 g/L COD loading, the CDM was 6.76 ± 0.08 g/L VSS from FWP 1, and it was 2.67 ± 0.86 g/L VSS from FWP 2. The PHBV yielded from FWP 1 was also much higher than FWP 2: with 40 g/L COD loading, the PHBV was 4.01 ± 0.05 g/L from FWP 1, and it was 0.67 ± 0.21 g/L from FWP 2. This may be because total carboxylates accounted for a higher portion of COD in FWP 2 than in FWP 1, which led to higher loadings of some carboxylic species from FWP 2 than in FWP 1 with a fixed COD loading. Some VFA species with high concentrations can be inhibitory to the cell growth of *H. mediterranei* [11]. Another reason can be the higher contents of other nutrients, including N, P, and the rest of COD besides carboxylates, which may boost cell growth and PHBV synthesis.

A general trend was observed that the CDM and PHBV production first increased and later decreased with higher substrate loadings, possibly due to substrate inhibition. The highest production also differed depending on feedstock types. For FWP 1, the highest CDM and PHBV production was 6.80 ± 0.08 and 4.00 ± 0.05 g/L, respectively, which were obtained from a substrate loading of 40 g/L COD. The yield from 40 g/L COD was 0.3 ± 0.0 g PHBV/g COD. For FWP 2, the highest CDM and PHBV production were 4.4 ± 0.4 and 2.3 ± 0.1 g/L, respectively, which were from a substrate loading of 60 g/L COD. 

Figure 4b,c demonstrates the PHBV content of cells and HV content of PHBV produced from different feedstocks with four substrate loadings. When FWP 1 was used, the loading levels did not influence the HV content and PHBV content with 40 to 80 g/L COD loadings. However, the substrate loading levels resulted in significantly different PHBV contents (*p*-value was 5.36 × 10^−6^) and HV contents (*p*-value was 3.45 × 10^−4^) when FWP 2 was used. FWP 1 resulted in higher cell contents of PHBV and HV content among all substrate loadings than FWP 2. For instance, with 40 g/L COD loading, the cell content of PHBV and HV content was 59.2 ± 0.1% and 16.5 ± 0.0% obtained from FWP 1, which were higher than 25.2 ± 0.2 and 13.0 ± 1.0% from FWP 2. A high cell content of PHBV suggests a high mass portion of CDM accounted for PHBV, indicating a better efficiency of PHBV synthesis. For FWP 1, the cell contents of PHBV were stable within 55% to 60% with all substrate loadings; however, for FWP 2, the cell contents of PHBV were within a wider range (5% to 50%). A high HV content of PHBV polymer suggests a high mass ratio of HV monomer versus HB monomer, which would benefit the flexibility and elasticity of bioplastic material. The HV contents derived from FWP 1 were at 16% to 18% of PHBV, and the HV contents derived from FWP 2 were below 16% of PHBV with all substrate loadings. 

The consumption of carboxylates is demonstrated in Figure 4d,e. Compared to FWP 2, there was more Pr consumed in FWP 1. For instance, with 40 g/L COD loading, cells consumed 6.87 ± 0.05 g/L Pr from FWP 1, and only 1.90 ± 0.42 g/L Pr was consumed from FWP 2 with the same substrate loading. Since Pr is the direct precursor to synthesize HV units of the PHBV, it can explain why the HV content from FWP 1 was higher than FWP 2. The HV content from FWP 1 was 16.5 ± 0.0%, while it was 13.0 ± 1.0% from FWP 2 with the same 40 g/L COD loading. These results suggest FWP 1 was a better candidate than FWP 2 because of higher CDM and PHBV production, cell content of PHBV, and higher HV content of PHBV polymers. A substrate loading of 40 g/L COD was selected among all other loadings for its higher production yields of CDM and PHBV.

### 3.4. Batch Production in Integrated Bioreactor System

**Figure 5:**
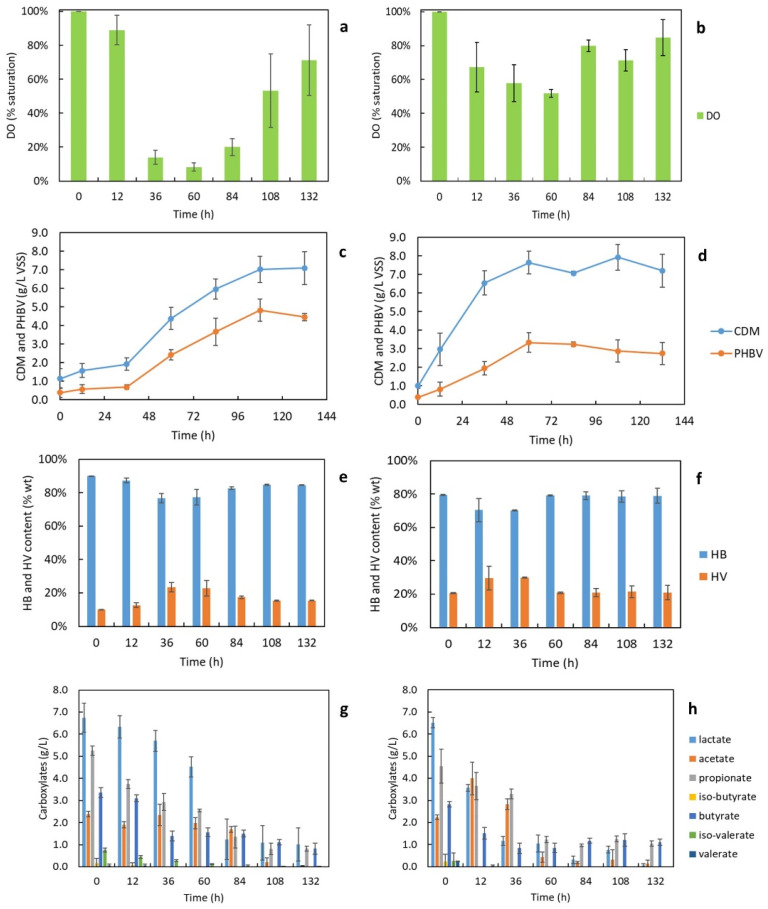
Time profiles in 6-L batch on (**a**) DO with an aeration rate of 2.5 vvm; and (**b**) with a controlled DO level above 50% sat. (**c**) Cell growth and PHBV production with an aeration rate of 2.5 vvm; and (**d**) with a controlled DO level above 50% sat. (**e**) Contents of 3-hydroxyvalerate (HV) and 3-hydroxybutyrate (HB) with an aeration rate of 2.5 vvm; and (**f**) with a controlled DO level above 50% sat. (**g**) Carboxylic consumption with an aeration rate of 2.5 vvm; and (**h**) with a controlled DO level above 50% sat.

As shown in Figure 5a, the DO of cell broth with a stable aeration rate of 2.5 vvm dropped abruptly from over 80% sat. to less than 20% sat. at the exponential growth phase when the cells were rapidly propagating (36 to 84 h). The DO increased back to over 60% sat. when the cells were approaching the stationary phase (108 h). With a different aeration strategy where the aeration rate was adjusted according to the DO level (Figure 5b), the DO of cell broth was maintained at over 50% sat. during the entire cultivation period.

The curves of cell growth and PHBV synthesis are shown in Figure 5c,d, which correspond to the two different aeration strategies. With a controlled aeration rate of 2.5 vvm, the concentration of CDM reached 7.0 ± 0.7 g/L VSS within 120 h and remained stable afterward. The PHBV concentration increased to 4.8 ± 0.6 g/L within the same culturing time. The two curves are approximately parallel to each other throughout the entire production, which aligns well with a previous finding that the PHBV synthesis from food waste-derived nutrients is a growth-associated reaction by *H. mediterranei* [5]. With a controlled DO level above 50% sat., the cells grew much faster and reached the stationary growth phase within 72 h, which shortened the cultivation period by 2 days. The final CDM (7.2 ± 0.9 g/L VSS) was close to the one with a controlled aeration rate, indicating that the second aeration strategy increased the productivity of CDM. However, the final PHBV was 2.8 ± 0.6 g/L, much lower than the one with a controlled aeration rate at 2.5 vvm (4.8 ± 0.6 g/L PHBV). This suggests a high DO level decreased the PHBV content of cells, which reduced PHBV production.

The time profiles of the HV and HB contents of PHBV produced with different aeration strategies are shown in Figure 5e,f. With a controlled aeration rate of 2.5 vvm, the HV content increased from 10.0 ± 0.1% wt. to 23.4 ± 2.9% wt. during the exponential growth phase, then decreased to around 15.5 ± 0.2% wt. at the stationary phase. With a controlled DO level above 50% sat., the HV content showed a similar trend: it increased to 29.8 ± 0.4% wt. during the exponential growth phase and dropped to around 21.0 ± 4.4% wt. in the stationary phase. The final HV content obtained from the batches with a DO above 50% sat. was higher than the ones with lower DO levels during the log phase. Therefore, although higher DO levels reduced PHBV production, as discussed previously, it led to higher HV content of PHBV, which may benefit the thermal and mechanical properties of the bioplastic product.

The consumption of short-chain carboxylates during cell growth is demonstrated in Figure 5g,h. The final consumptions of carboxylates were (average% of initial): 85% La, 98% Ac, 84% Pr, 76% Bu, and 100% Va in the batches with a controlled aeration rate of 2.5 vvm; and 99% La, 94% Ac, 77% Pr, 61% Bu, and 100% Va in the batches with DO levels above 50% saturation. In general, the consumption rates of the major carboxylates were much faster in the batches with DO levels above 50% sat. than in the batches with a controlled aeration rate at 2.5 vvm. Most of the carboxylates were consumed within 108 h for the batches with a controlled aeration rate of 2.5 vvm and within 60 h for the batches with a DO above 50% sat. Since the carboxylates account for most carbon sources in FWP, they were quickly utilized for faster cell growth, and PHBV synthesis with higher DO levels.

### 3.5. Effect of Spent Saline Medium Recycling

**Figure 6:**
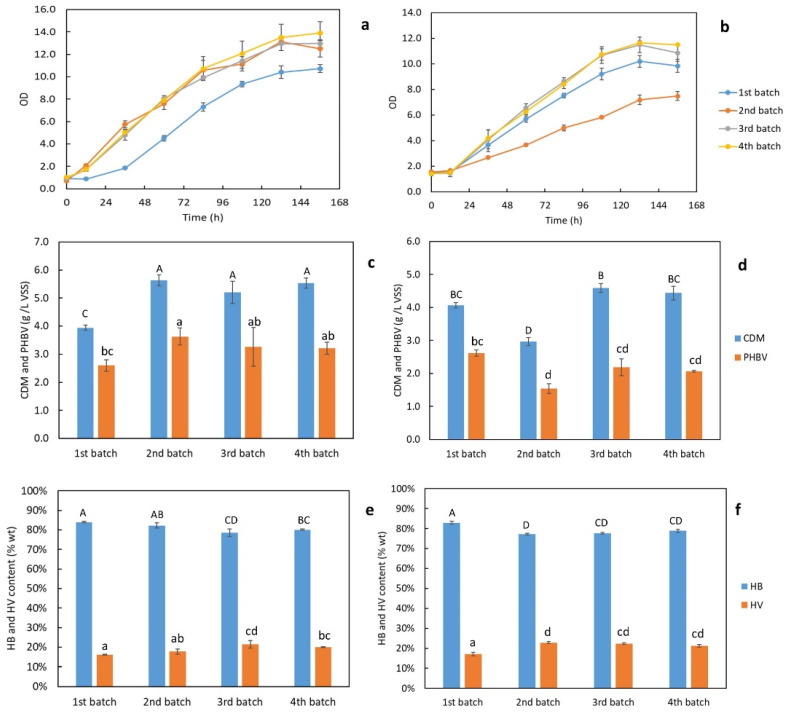
Four batches with spent salts recycled after H_2_O_2_ treatment: (**a**) growth curves with 80% spent salts recycled and; (**b**) with 90% spent salts recycled. CDM and PHBV productions (**c**) with 80% spent salts recycled and; (**d**) with 90% spent salts recycled. HB and HV contents of PHBV (**e**) with 80% spent salts recycled and; (**f**) with 90% spent salts recycled. Note: Same letters indicated that the mean values were not significantly different (*p* > 0.05).

Two series of SSM recycling experiments were conducted with 80% and 90% mass of SSM being recycled after H_2_O_2_ treatment in four consecutive batches, respectively. The cell growth curves shown in Figure 6a,b suggest that the cells grew faster and achieved higher final densities in the batches with 80% SSM recycling than the initial batch; similar growth was found in the batches with 90% SSM recycling except for the second batch. The results of the CDM and PHBV productions demonstrated in Figure 6c,d aligned well with the growth curves. With 80% SSM being recycled, the CDM produced in the 2nd, 3rd, and 4th recycled batches were 5.6 ± 0.2, 5.2 ± 0.4, and 5.5 ± 0.2 g/L, which were significantly higher than in the original batch (3.9 ± 0.1 g/L). Regarding PHBV production, only the second recycling batch (3.6 ± 0.3 g/L PHBV) was significantly higher than the original batch (2.6 ± 0.2 g/L PHBV). The remaining recycling batches, which were 3.3 ± 0.7 g/L PHBV from the 3rd batch and 3.2 ± 0.2 g/L PHBV from the 4th batch, had similar productions to the original one (the differences were not significant). With the 90% SSM being recycled, the CDM (3.0 ± 0.1 g/L) and PHBV (1.5 ± 0.2 g/L) productions in the second batch were significantly lower than the original batch (4.1 ± 0.1 g/L CDM and 2.6 ± 0.1 g/L PHBV). The PHBV productions from the third (2.2 ± 0.3 g/L) and fourth batches (2.1 ± 0.0 g/L) were not significantly different from the first batch. The reason for the lower CDM and PHBV productions in the second batch could be the inhibitory compounds in the H_2_O_2_-treated SSM, such as leftover COD or H_2_O_2,_ that limited the cell growth. However, the cells were able to acclimate to this condition quickly and regained normal growth and PHBV synthesis in the following recycling batches. The contents of HV of PHBV, illustrated in Figure 6e,f, increased slightly from 16.1 ± 0.3% to 20.0 ± 0.3% in the batches where 80% SSM was being recycled and from 17.1 ± 0.8% to 21.2 ± 0.7% in the batches with 90% SSM being recycled. With 80% of SSM recycling, the HV contents of the PHBV products were higher in the third and fourth batches; and with 90% of SSM recycling, the HV contents were higher in all recycling batches. Therefore, in addition to the relatively stable productions of CDM and PHBV, it can also increase the HV content of the PHBV products. The higher HV content of PHBV can introduce more flexibility and elasticity to the thermoplastic material, which can help broaden the product applications.

Both the 80% and 90% SSM recycling experiments suggest a promising potential of employing the H_2_O_2_ pretreatment strategy on the SSM to maintain normal cell growth and PHBV synthesis from food waste with the use of spent salts. The recycling of SSM can provide economic and environmental benefits due to the major savings of purchasing new salts and the costly treatment and disposal of high-saline wastewater. Another advantage is the possibility of achieving better product quality through a significant increase in HV content. Based on the findings of this study, a further research interest can be the optimization of chemical treatment and recycling conditions in a pilot to commercial production scale and demonstrating the feasibility and economic efficacy for practical applications.

## 4. Discussion

Fermented food waste can provide sufficient sources of carbon, nitrogen, and phosphorous, with abundant essential nutrients and trace elements to sustain the microbial growth of *H. mediterranei* for PHBV synthesis. Only additional sodium and magnesium salts were needed to provide enough salinity. Therefore, it would result in a reduced production cost because food waste is an inexpensive feedstock with additional tipping fees for treatment, and the usage of salts is much less [4,35,36]. This study provides a yield of 0.3 g PHBV/g COD, which is comparable to the yields reported by other studies [5,14,17,37,38]. 

To address the hot topic of saline wastewater disposal, a novel salt recovery and recycling process has been developed to minimize salt discharge in the system. The recycling of 90% spent salts has been successfully achieved for four consecutive batches after the treatment by H_2_O_2_. The final PHBV production after four recycling batches was 2.1 ± 0.0 g/L, which was close to the original batch (2.6 ± 0.0 g/L). The contents of HV and HB from the last batch were 21.2 ± 0.7% and 78.8 ± 0.7%, which were also similar to those obtained from the original batch (17.1 ± 0.8% and 82.9 ± 0.8%). These results indicate that recycling spent salts after pretreatment can provide robust PHBV production from fermented food waste and consistent product qualities. This offers new sustainable insights into the current research area focusing on halophilic PHA producers and will further benefit the systematic applicability and profitability.

Besides, the study found that the dynamic dissolved oxygen levels throughout microbial cultivation were critical in terms of influencing microbial growth and PHBV synthesis rates, product yield and quality, and metabolic patterns. This DO effect can be further investigated as a lever to customize the product composition for specific needs in biomaterial thermal and mechanical properties.

As one promising inexpensive feedstock, food waste has variable source compositions and qualities, even for those collected from the same food waste processing facility. These variables introduce uncertainty and instability to the PHA production processes, which could potentially risk good manufacturing practices in commercial-scale production. Therefore, it is necessary to identify the influence of food waste complexity on PHA production and optimize the feedstock through viable and economic approaches. Another issue is the uncertainty of the short-chain carboxylates profile derived from the anaerobic fermentation process. As the precursors to PHBV synthesis, the profile of carboxylates can significantly influence product yield and quality. Therefore, further research is needed to find ways to manipulate the anaerobic fermentation process that give the optimum carboxylate profile to PHA production.

## 5. Conclusions

This study developed a sustainable bioprocessing system integrating biodegradable plastic production and the valorization of municipal food waste. Food waste permeates with optimum levels (40 g/L COD loading and 2.5 vvm of aeration) yielded 0.3 g PHBV/g COD. A higher DO increases the growth rate but lowers the PHA production. With H_2_O_2_ treatment and evaporation, 90% of the spent salts were successfully reused for four consecutive batches and yielded consistent PHA concentrations and product qualities. The production system has the potential for practical applications in bridging current food and bioplastic industries and provides economic incentives to improve organic waste management.

## Figures and Tables

**Table 1:** Characterization of nutrients in the streams of the microfiltration process.

		Food Waste 1			Food Waste 2		
Compound	Unit	Influent 1	Permeate 1	Retentate 1	Influent 2	Permeate 2	Retentate 2
Lactic acid	g/L	24.7 ± 0.9	21.5 ± 1.3	20.4 ± 0.6	37.8 ± 0.4	36.6 ± 0.5	36.0 ± 0.4
Acetic acid	g/L	10.1 ± 0.8	8.7 ± 0.6	8.3 ± 0.4	17.7 ± 0.2	16.8 ± 0.1	15.9 ± 0.1
Propionic acid	g/L	20.0 ± 1.2	17.2 ± 1.1	16.7 ± 0.6	14.1 ± 0.2	12.9 ± 0.2	12.2 ± 0.1
Butyric acid	g/L	4.4 ± 0.3	3.8 ± 0.3	2.8 ± 0.1	4.4 ± 0.1	4.1 ± 0.1	3.6 ± 0.1
Iso-butyric acid	g/L	0.4 ± 0.0	0.4 ± 0.0	0.4 ± 0.0	0.3 ± 0.0	0.3 ± 0.0	0.3 ± 0.0
Iso-valeric acid	g/L	0.7 ± 0.0	0.6 ± 0.0	0.7 ± 0.1	1.0 ± 0.1	0.9 ± 0.1	0.8 ± 0.0
Valeric acid	g/L	0.1 ± 0.0	0.1 ± 0.0	0.3 ± 0.0	0.1 ± 0.0	0.0 ± 0.0	0.0 ± 0.0
Total carboxylic acids	g/L	60.2 ± 3.5	52.4 ± 3.4	49.7 ± 2.8	76.2 ± 0.5	71.6 ± 0.7	68.8 ± 0.4
COD from acids	g/L	82.5 ± 1.5	71.5 ± 1.7	71.1 ± 2.0	96.5 ± 1.0	84.8 ± 0.5	83.9 ± 0.5
Total COD	g/L	232.6 ± 1.5	108.5 ± 2.0	560.3 ± 7.3	108.5 ± 0.8	92.5 ± 1.0	149.8 ± 1.2
Total N	g/L N	4.6 ± 0.3	3.8 ± 0.1	6.7 ± 0.4	3.0 ± 0.2	2.5 ± 0.1	4.3 ± 0.3
Total P	g/L P	3.7 ± 0.1	0.8 ± 0.0	6.5 ± 0.1	2.8 ± 0.1	0.6 ± 0.0	4.8 ± 0.2

**Table 2:** Nutrient elements in fermented food waste permeate.

Compound	Unit	Permeate 1	Permeate 2	MSM and SL-6
N	g/L	3.8 ± 0.1	2.5 ± 0.1	0
P	g/L	0.8 ± 0.0	0.6 ± 0.0	0
K	mg/L	2055 ± 7	2072 ± 5	1950
Ca	mg/L	1588 ± 3	1629 ± 6	183
Mg	mg/L	202 ± 0	198 ± 2	5346
Na	mg/L	1579 ± 2	1485 ± 1	61,410
B	mg/L	1.69 ± 0.00	1.62 ± 0.01	0.5
Zn	mg/L	3.79 ± 0.01	3.83 ± 0.01	0.25
Cu	mg/L	0.50 ± 0.01	0.38 ± 0.00	0.04
Mn	mg/L	2.81 ± 0.00	2.92 ± 0.01	0.13
Fe	mg/L	30.80 ± 0.71	30.52 ± 0.65	17.3
Ni	mg/L	0.20 ± 0.00	0.18 ± 0.00	0.05

## Data Availability

Datasets related to this article can be found at https://data.mendeley.com/datasets/r38mjyvfgr, an open-source online data repository hosted at Mendeley Data [39].

## References

[1] Reddy C.S.K., Ghai R., Rashmi, Kalia V.C. (2003). Polyhydroxyalkanoates: An Overview. Bioresour. Technol..

[2] Chen G.Q. (2009). A Microbial Polyhydroxyalkanoates (PHA) Based Bio- and Materials Industry. Chem. Soc. Rev..

[3] Nielsen C., Rahman A., Rehman A., Walsh M., Miller C. (2017). Food Waste Conversion to Microbial Polyhydroxyalkanoates. Microb. Biotechnol..

[4] Wang K., Hobby A.M., Chen Y., Chio A., Jenkins B.M., Zhang R. (2022). Techno-Economic Analysis on an Industrial-Scale Production System of Polyhydroxyalkanoates (PHA) from Cheese By-Products by Halophiles. Processes.

[5] Wang K., Zhang R. (2021). Production of Polyhydroxyalkanoates (PHA) by *Haloferax mediterranei* from Food Waste Derived Nutrients for Biodegradable Plastic Applications. J. Microbiol. Biotechnol..

[6] Campanari S., Augelletti F., Rossetti S., Sciubba F., Villano M., Majone M. (2017). Enhancing a Multi-Stage Process for Olive Oil Mill Wastewater Valorization towards Polyhydroxyalkanoates and Biogas Production. Chem. Eng. J..

[7] Colombo B., Pepè Sciarria T., Reis M., Scaglia B., Adani F. (2016). Polyhydroxyalkanoates (PHAs) Production from Fermented Cheese Whey by Using a Mixed Microbial Culture. Bioresour. Technol..

[8] Liu H.-Y., Hall P.V., Darby J.L., Coats E.R., Green P.G., Thompson D.E., Loge F.J. (2008). Production of Polyhydroxyalkanoate During Treatment of Tomato Cannery Wastewater. Water Environ. Res..

[9] Shen L., Hu H., Ji H., Cai J., He N., Li Q., Wang Y. (2014). Production of Poly(Hydroxybutyrate–Hydroxyvalerate) from Waste Organics by the Two-Stage Process: Focus on the Intermediate Volatile Fatty Acids. Bioresour. Technol..

[10] Bhattacharyya A., Jana K., Haldar S., Bhowmic A., Mukhopadhyay U.K., De S., Mukherjee J. (2015). Integration of Poly-3-(Hydroxybutyrate-Co-Hydroxyvalerate) Production by *Haloferax mediterranei* through Utilization of Stillage from Rice-Based Ethanol Manufacture in India and Its Techno-Economic Analysis. World J. Microbiol. Biotechnol..

[11] Ferre-Guell A., Winterburn J. (2018). Biosynthesis and Characterization of Polyhydroxyalkanoates with Controlled Composition and Microstructure. Biomacromolecules.

[12] Quillaguamán J., Guzmán H., Van-Thuoc D., Hatti-Kaul R. (2010). Synthesis and Production of Polyhydroxyalkanoates by Halophiles: Current Potential and Future Prospects. Appl. Microbiol. Biotechnol..

[13] Wang J., Yue Z.-B., Sheng G.-P., Yu H.-Q. (2010). Kinetic Analysis on the Production of Polyhydroxyalkanoates from Volatile Fatty Acids by Cupriavidus Necator with a Consideration of Substrate Inhibition, Cell Growth, Maintenance, and Product Formation. Biochem. Eng. J..

[14] Ghosh S., Gnaim R., Greiserman S., Fadeev L., Gozin M., Golberg A. (2019). Macroalgal Biomass Subcritical Hydrolysates for the Production of Polyhydroxyalkanoate (PHA) by *Haloferax mediterranei*. Bioresour. Technol..

[15] DEP DO Saturation Calculator|Florida Department of Environmental Protection. https://floridadep.gov/dear/water-quality-standards-program/documents/do-saturation-calculator%C2%A0.

[16] Cui Y.-W., Shi Y.-P., Gong X.-Y. (2017). Effects of C/N in the Substrate on the Simultaneous Production of Polyhydroxyalkanoates and Extracellular Polymeric Substances by *Haloferax mediterranei* via Kinetic Model Analysis. RSC Adv..

[17] Pais J., Serafim L.S., Freitas F., Reis M.A.M. (2016). Conversion of Cheese Whey into Poly(3-Hydroxybutyrate-Co-3-Hydroxyvalerate) by *Haloferax mediterranei*. New Biotechnol..

[18] Lillo J.G., Rodriguez-Valera F. (1990). Effects of Culture Conditions on Poly(3-Hydroxybutyric Acid) Production by *Haloferax mediterranei*. Appl. Environ. Microbiol..

[19] Koller M., Hesse P., Bona R., Kutschera C., Atlić A., Braunegg G. (2007). Biosynthesis of High Quality Polyhydroxyalkanoate Co- and Terpolyesters for Potential Medical Application by the Archaeon *Haloferax mediterranei*. Macromol. Symp..

[20] Koller M. (2015). Recycling of Waste Streams of the Biotechnological Poly(Hydroxyalkanoate) Production by *Haloferax mediterranei* on Whey. Int. J. Polym. Sci..

[21] Lorantfy B., Seyer B., Herwig C. (2014). Stoichiometric and Kinetic Analysis of Extreme Halophilic Archaea on Various Substrates in a Corrosion Resistant Bioreactor. New Biotechnol..

[22] U.S. Environmental Protection Agency Indicators: Salinity. https://www.epa.gov/national-aquatic-resource-surveys/indicators-salinity.

[23] U.S. Environmental Protection Agency Water Quality Standards: Regulations and Resources. https://www.epa.gov/wqs-tech.

[24] Bhattacharyya A., Saha J., Haldar S., Bhowmic A., Mukhopadhyay U.K., Mukherjee J. (2014). Production of Poly-3-(Hydroxybutyrate-Co-Hydroxyvalerate) by *Haloferax mediterranei* Using Rice-Based Ethanol Stillage with Simultaneous Recovery and Re-Use of Medium Salts. Extremophiles.

[25] Chen Y., Liu C., Nie J., Wu S., Wang D. (2014). Removal of COD and Decolorizing from Landfill Leachate by Fenton’s Reagent Advanced Oxidation. Clean Technol. Environ. Policy.

[26] Zaharia C., Suteu D., Muresan A., Muresan R., Popescu A. (2009). Textile Wastewater Treatment by Homogenous Oxidation with Hydrogen Peroxide. Environ. Eng. Manag. J..

[27] Zhang R., El-Mashad H.M., Hartman K., Wang F., Liu G., Choate C., Gamble P. (2007). Characterization of Food Waste as Feedstock for Anaerobic Digestion. Bioresour. Technol..

[28] Dyall-Smith M. (2009). The Halohandbook Protocols for Halobacterial Genetics.

[29] Sluiter A., Hames B., Ruiz R., Scarlata C., Sluiter J., Templeton D. (2008). Determination of Sugars, Byproducts, and Degradation Products in Liquid Fraction Process Samples: Laboratory Analytical Procedure (LAP).

[30] APHA (2012). Standard Methods for the Examination of Water and Wastewater.

[31] Escalona A.M., Varela F.R., Gomis A.M. (1996). Procedure for Extraction of Polyhydroxyalkanoates from Halophilic Bacteria Which Contain Them. U.S. Patent.

[32] Yu J., Si Y., Keung W., Wong R. (2002). Kinetics Modeling of Inhibition and Utilization of Mixed Volatile Fatty Acids in the Formation of Polyhydroxyalkanoates by Ralstonia Eutropha. Process Biochem..

[33] Doran P.M. (2013). Bioprocess Development. Bioprocess Engineering Principles.

[34] Garcia-Ochoa F., Gomez E. (2009). Bioreactor Scale-up and Oxygen Transfer Rate in Microbial Processes: An Overview. Biotechnol. Adv..

[35] Choi J., Lee S.Y. (1997). Process Analysis and Economic Evaluation for Poly(3-Hydroxybutyrate) Production by Fermentation. Bioprocess Eng..

[36] Leong Y.K., Show P.L., Lan J.C.-W., Loh H.-S., Lam H.L., Ling T.C. (2017). Economic and Environmental Analysis of PHAs Production Process. Clean Technol. Environ. Policy.

[37] Alsafadi D., Al-Mashaqbeh O. (2017). A One-Stage Cultivation Process for the Production of Poly-3-(Hydroxybutyrate-Co-Hydroxyvalerate) from Olive Mill Wastewater by *Haloferax mediterranei*. New Biotechnol..

[38] Ferre-Guell A., Winterburn J. (2017). Production of the Copolymer Poly(3-Hydroxybutyrate-Co-3-Hydroxyvalerate) with Varied Composition Using Different Nitrogen Sources with *Haloferax mediterranei*. Extremophiles.

[39] Wang K. (2022). Process Development of Polyhydroxyalkanoates Production by Halophiles Valorizing Municipal Food Waste with Spent Salts Recycling: Datasets and Supplemental Materials. https://data.mendeley.com/datasets/r38mjyvfgr.

